# Supplementary material for: Genetic diversity, haplotype analysis, and risk factor assessment of hepatitis A virus isolates from the West Bank, Palestine during the period between 2014 and 2016
Source: PLoS One. 2020 Dec 11;15(12):e0240339. doi: 10.1371/journal.pone.0240339 (PMC7732126; doi:10.1371/journal.pone.0240339)
Supplement: S1 Questionnaire — (DOCX) [file pone.0240339.s001.docx]

| **Hepatitis A questionnaire** | | | | |
| --- | --- | --- | --- | --- |
| Patient Name |  | | Date of HAV infection: | ……../……/…. |
| DOB | …../……../……. |  | Sex | Male Female |
| Marital status | Single | Married | divorce |  |
| Address | City/town/camp: ................................ | | District: ............................................. | |
| Level of education | Elementary school | Middle school | University |  |
| Occupation |  | | | |
| Income (ILS) | < 1450 | 1450- 3000, | 3000-5000 | >5000 |
| **Clinical history** | | | | |
| Have you ever had surgical procedure? | Yes | No |  |  |
| If the answer is yes, What type of surgical procedure did you do | …………………………………………………………………………………………. | | | |
| Do you receive blood transfusion | Yes | No |  |  |
| If the answer is yes, when and where |  | | | |
| Have you ever had a dentist procedures? | Yes | No |  |  |
| Have you ever had jaundice? | Yes | No |  |  |
| Are you infected with HCV? | Yes | No |  |  |
| Are you infected with HBV? | Yes | No |  |  |
| Do you have a household contact with HBV and/or HCV | Yes | No |  |  |
| Have you ever been abroad? | Yes | No |  |  |
| **Personal hygiene:** | | | | |
| Toilette | in house | in yard |  |  |
| Type of toilette | flush, | open pits, | bucket |  |
| Drinking water source | Well | piped into house | piped into yard | spring |
| If not pipe lined, do you treat water before drinking? | Yes | No | How: ……………………………… | |
| How often do you wash hands after defecation | Every time | some times |  |  |
| How often do you wash hands before lunch | Every time | some times |  |  |
| Eat raw vegetables | Yes | No |  |  |
| Eat unpeeled fruits | Yes | No |  |  |
| Swimming practice | Yes | No |  |  |
| Eating out side home | Yes | No |  |  |
| If yes, where | …………………………………………………………………………………………… | | | |
| Do you raise domestic animals? | Yes | No |  |  |
|  | Yes, mention………. | | | |
|  | No, have animals in vicinity | | Yes | No |
| **Lab test results** | | | | |
| HAV IgM | Positive | Negative |  |  |
| RT-PCR/serum | Positive | Negative |  |  |
|  |  |  |  |  |
