# Supplementary material for: Genetic diversity, haplotype analysis, and risk factor assessment of hepatitis A virus isolates from the West Bank, Palestine during the period between 2014 and 2016
Source: PLoS One. 2020 Dec 11;15(12):e0240339. doi: 10.1371/journal.pone.0240339 (PMC7732126; doi:10.1371/journal.pone.0240339)
Supplement: S2 Questionnaire — (DOCX) [file pone.0240339.s002.docx]

| **استبانة بحث فيروس التهاب الكبد الوبائي نوع أ** | | | | |
| --- | --- | --- | --- | --- |
| .......................... | تاريخ الاصابة: |  | | الاسم |
| انثى | ذكر | الجنس | ......../........./.............. | تاريخ الولادة |
|  | مطلق/مطلة | متزوج/متزوجة | اعزب/عزباء | الحالة الاجتماعية |
|  | المحافظه.................................... | | مدينة/قرية/مخيم............. | العنوان |
|  | جامعي | ثانوي | اساسي | مستوى التعليم |
|  |  |  |  | المهنة |
| >5000 | 3000-5000 | 1400-3000 | < 1400 | الدخل (بالشيكل) |
| **التاريخ الطبي** | | | | |
|  |  | لا | نعم | هل سبق لك أن خضعت لعملية جراحية؟ |
| .................................................................................................................... | | | | إذا كان الجواب نعم ، فما نوع العملية الجراحية التي قمت بها؟ |
|  |  | لا | نعم | هل تلقيت نقل دم |
| ……………………………………………………………………....…… | | | | إذا كان الجواب نعم ، متى وأين? |
|  |  | لا | نعم | هل سبق لك أن خضعت لعلاج طبيب الأسنان؟ |
|  |  | لا | نعم | هل أصبت باليرقان من قبل؟ |
|  |  | لا | نعم | هل تعاني من التهاب الكبد الفيروسي نوع ت؟ |
|  |  | لا | نعم | هل تعاني من التهاب الكبد الفيروسي نوع ب؟ |
|  |  | لا | نعم | هل لديك اتصال باحد افراد اسرتك ممن يعانون من الإصابة بفيروس HBV و / أو HCV |
|  |  | لا | نعم | هل سافرت خارج البلاد من قبل؟ |
| **النظافة الشخصية** | | | | |
|  |  | بالفناء | داخل البيت | دورة المياه |
|  | دلو (وعاء) | حفرة مفتوحة | مرحاض دافق | نوع دورة المياه |
|  | نبع | شيكة مياه عامة | ابار منزلية | مصدر مياه الشرب |
| كيف............................................. | | لا | نعم | إن لم يكن مصدر المياه شبكة عامة، فهل تعالج الماء قبل الشرب؟ |
|  |  | بعض المرات | كل مرة | كم مرة تغسل يديك بعد التغوط |
|  |  | بعض المرات | كل مرة | كم مرة تغسل يديك قبل تناول الطعام |
|  |  | لا | نعم | هل تتناول خضروات نية |
|  |  | لا | نعم | هل تتناول فواكه غير مقشرة |
|  |  | لا | نعم | هل تمارس السباحة |
|  |  | لا | نعم | هل تاكل خارج البيت |
| .................................................................................................................... | | | | اذا كنت تاكل خارج البيت، اذكر المكان |
|  |  | لا | نعم | هل تربي الحيوانات الأليفة؟ |
| .................................................................................................................... | | | | اذا كان جواب السؤال السابق نعم، اذكر نوع الحيوان |
|  |  | لا | نعم | اذا كان الجواب لا، هل يوجد حيوانات في الجوار |
| **نتائج فحص المختبر** | | | | |
|  |  |  |  | HAV IgM |
|  |  |  |  | HAV-RT-PCR |
